# Complex heatmap visualization

**DOI:** 10.1002/imt2.43

**Published:** 2022-08-01

**Authors:** Zuguang Gu

**Affiliations:** ^1^ Molecular Precision Oncology Program, National Center for Tumor Diseases (NCT) Heidelberg Germany

**Keywords:** bioconductor, clustering, complex heatmap, R package, visualization

## Abstract

Heatmap is a widely used statistical visualization method on matrix‐like data to reveal similar patterns shared by subsets of rows and columns. In the R programming language, there are many packages that make heatmaps. Among them, the *ComplexHeatmap* package provides the richest toolset for constructing highly customizable heatmaps. *ComplexHeatmap* can easily establish connections between multisource information by automatically concatenating and adjusting a list of heatmaps as well as complex annotations, which makes it widely applied in data analysis in many fields, especially in bioinformatics, to find hidden structures in the data. In this article, we give a comprehensive introduction to the current state of *ComplexHeatmap*, including its modular design, its rich functionalities, and its broad applications.

## Highlights

- Complex heatmap is a powerful visualization method for revealing associations between multiple sources of information.
- We have developed an R package named *ComplexHeatmap* that provides comprehensive functionalities for heatmap visualization. It has been widely used in the bioinformatics community.
- We give a comprehensive introduction to the current state of *ComplexHeatmap* in this article.

## Graphical abstract

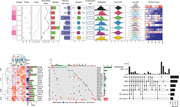

## INTRODUCTION

Heatmap is a popular method for visualizing matrix‐like data by taking colors as the aesthetic elements. There are two major categories of heatmap visualization: spatial heatmap and grid heatmaps [1]. The first category visualizes spatially distributed patterns, such as the global temperature distribution across the world, or the click activities on a web page from users. The so‐called choropleth map also utilizes heatmaps to visualize certain characteristics in geographic areas within a region. The second category is purely a rectangular layout of colored grids, where the two dimensions correspond to two types of variables. In most cases, rows and columns of a heatmap are reordered by certain methods so that subsets of rows and columns showing similar patterns are grouped closely on the heatmap. The reordering is mostly applied by hierarchical clustering, thus the grid heatmap is also called the cluster heatmap. In this article, we only discuss the grid heatmap.

Heatmap visualization can be traced back to the 19th century when it was used to visualize various social statistics in different districts in Paris [2]. However, as a statistical visualization method, it has only been widely used when it was applied in bioinformatics since the 1990s. Since the very early paper on heatmap visualization on gene expression datasets was published in 1998 [3], heatmap has been a standard tool for visualizing omics‐level data, for example, gene expression or DNA methylation datasets that are represented as matrices. Nowadays, heatmap is applied in genomics for more specific studies, for example, to visualize genome‐level regulation on three‐dimensional (3D) scales [4], DNA methylation signals in the binned genome [5], or enrichment of a certain type of genomic signals around a specific genomic feature [6]. Rectangular layout is the most used for heatmap visualization; moreover, there are also other layouts, such as the circular layout [7], the spiral layout [8], and the Hilbert curve layout [9]. They are useful in specific scenarios.

R is a popular programming language for data analysis and visualization. In R, there are a number of packages that make heatmaps. The function heatmap() from the *stats* package is the most fundamental one, but with very limited functionality. The function heatmap.2() from the *gplots* package is an enhanced version of heatmap() which supports more graphics on heatmaps, such as color legends with value distributions and trace lines showing the difference of values to the column or row medians. The function geom_tile() from the *ggplot2* package [10] also provides a simple implementation of heatmaps. There are also packages that provide more flexible controls on heatmaps, such as the function pheatmap() from the *pheatmap* package, and the function aheatmap() from the *NMF* package [11].

As data emerge fast in sizes and dimensions nowadays, especially in the field of genomics, an efficient visualization for integrative analysis or multi‐omics analysis is urgently needed to associate multiple types of data for easily revealing relationships between multiple objects. From the aspect of heatmap visualization, it reflects two points. The first one is the support of heatmap annotations, which contain extra information to associate with the main heatmap. For example, in a typical heatmap visualization on gene expression data where rows are genes and columns are patients, it is common that patients have clinical metadata available, such as age, gender, or whether the patient has certain DNA mutations. With annotations attached to the heatmap, it is easy to identify, for example, whether a group of genes showing high expression correlates to a certain age interval, or whether they have specific types of DNA mutations. heatmap() and heatmap.2() only support a single heatmap‐like annotation for one numeric or character vector. pheatmap() and aheamtap() allow multiple heatmap‐like annotations for corresponding more information to the heatmap. The *superheat* [12] and *heatmap3* [13] packages support more types of graphics for annotations, such as points or lines that are able to make more accurate visual representations of the data. The second point of visualizing multiple sources of information is to directly apply “complex heatmap visualization” by simultaneously linking multiple heatmaps to make it straightforward to compare patterns shared between heatmaps. For example, in our previous study [6], we applied complex heatmap visualization on data of gene expression, DNA methylation, and various histone modifications to reveal general transcriptional regulation patterns among multiple human tissues. To implement both complex annotation and heatmap visualization, we have developed an advanced heatmap package named *ComplexHeatmap* [14]. It supports not only the basic annotation graphics as in other packages, but also a variety of extra complex annotation graphics such as violin plot or horizon chart, and it even allows users to self‐define their own annotation graphics. *ComplexHeatmap* provides a simple syntax to link multiple heatmaps and annotations where rows or columns of all heatmaps are adjusted simultaneously. The simplicity of its user interface and comprehensiveness of its functionalities make *ComplexHeatmap* widely used in the bioinformatics community to reveal interesting patterns from data that are potentially biologically meaningful.

The *ComplexHeatmap* project was started in 2015 and the corresponding paper was published in 2016 [14]. Since then, it has become a popular tool in the bioinformatics field. It has been downloaded more than 500k times and 104 other CRAN/Bioconductor packages have direct dependencies on it (data collected on June 22, 2022). *ComplexHeatmap* has been applied to build comprehensive visualization in a wide range of biology studies, such as on cancers [15], COVID‐19 [16], single cells [17], immunology [18], as well as in other fields, such as oceanology [19] and ecology [20]. Continually in the past 6 years, *ComplexHeatmap* has been actively maintained with many new features added. We have also reformatted the documentation as a comprehensive book (https://jokergoo.github.io/ComplexHeatmap-reference/book/). In this article, we will give a comprehensive introduction on the current state of *ComplexHeatmap*, including its modular design, its rich functionalities, and its broad applications.

## RESULTS AND DISCUSSION

### Modular design

*ComplexHeatmap* is designed in a modular and object‐oriented way. There are three major classes defined in *ComplexHeatmap*: the *Heatmap* class that defines a complete heatmap with multiple components, the *HeatmapAnnotation* class that defines a list of annotations with specific graphics, and the *HeatmapList* class that manages a list of heatmaps and heatmap annotations.

**Figure 1:**
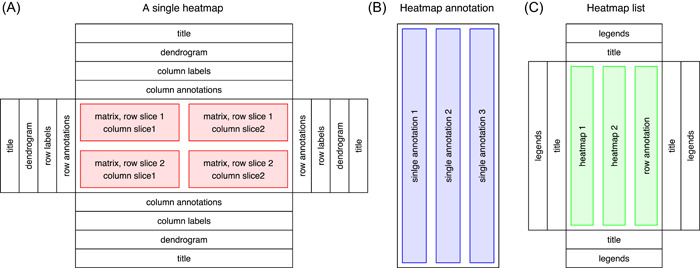
Modular design of the *ComplexHeatmap* package. (A) Single heatmap with various heatmap components. (B)  Heatmap annotation with a list of single annotations. (C)  Heatmap list with global titles and legends.

The heatmap is the basic unit of complex heatmap visualization. A single heatmap is composed of the heatmap body and various heatmap components (Figure 1A). The heatmap body is a two‐dimensional arrangement of grids where each grid corresponds to a specific value in the input matrix. The heatmap components contain titles, dendrograms, labels for matrix rows and columns, and heatmap annotations. These components can be optionally put on the four sides of the heatmap body and each component is managed by a specific method that is defined for the *Heatmap* object. Additionally, the heatmap body can be split into rows and columns, for example, by categorical variables, into slices. Dendrograms, heatmap labels, and annotations are then reordered or split accordingly.

Heatmap annotations contain additional information that associates with rows or columns of the heatmap. *ComplexHeatmap* provides rich support for setting different annotations and defining new annotation graphics. The annotations can be put on the four sides of the heatmap as its components, and they can also be arranged independently to be concatenated to the heatmaps. A *HeatmapAnnotation* object contains a list of single annotations, which are defined by the *SingleAnnotation* class (Figure 1B). Every single annotation contains a specific type of graphic, which is further defined by the *AnnotationFunction* class. The *AnnotationFunction* class provides a flexible way to define new annotation graphics, which can be automatically reordered and split according to the main heatmap.

The main feature of *ComplexHeatmap* is it supports concatenating a list of heatmaps and annotations horizontally or vertically to visualize associations between different data sources. The *HeatmapList* class is a container of a list of heatmaps and annotations (Figure 1C) and it automatically adjusts the correspondence of rows or columns in multiple heatmaps and annotations.

### Single heatmap

*ComplexHeatmap* provides rich functionalities for configuring single heatmaps. The constructor function Heatmap() makes a single heatmap and it returns an object in the *Heatmap* class. The only mandatory argument in Heatmap() is a matrix, either in numeric or character. Heatmap() provides a large number of additional arguments for customizing heatmaps. Besides the common functionalities also available in other heatmap packages, Heatmap() has these unique features listed in the following subsections.

#### Flexible controls of clustering and reordering

**Figure 2:**
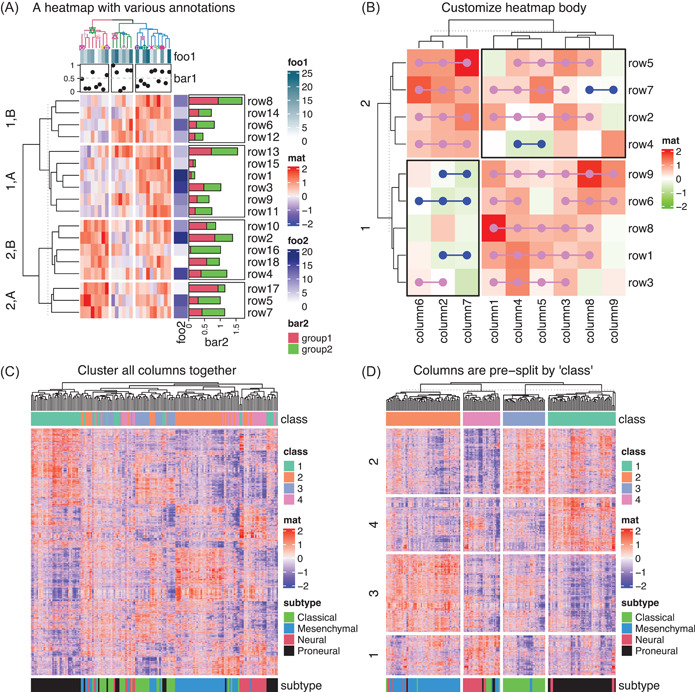
Demonstration of single heatmaps. (A) Heatmap with both row and column annotations. The columns on the heatmap are split by a three‐group *k*‐means clustering and rows are split by combinations of a categorical variable and a two‐group *k*‐means clustering. (B) Heatmap with customizations on the heatmap body. Data in Figure (A) and (B) are randomly generated. (C) Heatmap without and (D) with column splitting. The matrix for Figure (C) and (D) is the same.

In routine data analysis procedures, the matrix for heatmap visualization is normally accompanied by hierarchical clustering, so that features with similar patterns are grouped closely and they can be easily identified from the colors on heatmap. In Heatmap(), the hierarchical clustering can be specified in various ways: (1) by a predefined distance method, such as “euclidean” or “Pearson,” (2) by a distance function that calculates the pairwise distance between two vectors or directly from a matrix, (3) by a clustering function that takes a matrix as input and returns a *dendrogram* object, and (4) by a clustering object, for example, a *hclust* or a *dendrogram* object, or an object that can be coerced to a *dendrogram* object by a proper as.dendrogram() function. The last method is especially useful because it makes it possible to use dendrograms generated or edited by other packages. For example, with the *dendextend* package [21], dendrogram branches can be rendered with different colors to highlight sub‐dendrograms, or specific symbols can be added to dendrogram nodes, and then the rendered dendrograms can be seamlessly integrated with Heatmap() (Figure 2A).

A dendrogram is normally represented as a binary tree where the order of two branches is assigned arbitrarily on a node. Rotating the two branches on a node does not change its mathematical representation, but it affects the global ordering of dendrogram leaves. Thus, a proper way to rotate dendrogram branches, or in other words, to reorder the dendrogram, helps to move matrix rows or columns with similar patterns closer to each other in the heatmap to improve the visualization. By default, Heatmap() uses reorder.dendrogram() to reorder the dendrogram based on the mean of the submatrix for the dendrogram branches. For example, on every node in a dendrogram, one branch with a smaller mean value is always put on the left of the node. Heatmap() also accepts *dendrogram* objects, thus other dendrogram reordering methods can be easily integrated by first generating *dendrogram* objects for rows or columns, then applying a specific dendrogram reordering method, such as from the *dendsort* package [22], and finally sending them to Heatmap().

Note hierarchical clustering is just a special way to reorder rows and columns of a heatmap. Other methods that calculate row and column orders of a matrix can also be integrated. Heatmap() allows users to set numeric or character indices to reorder heatmaps. Examples of popular packages for ordering matrices are *seriation* [23] and *biclust* [24].

#### Split heatmap

Heatmap splitting is an efficient way of highlighting group‐wise patterns. Due to the hierarchical clustering procedure, when a new leaf or a sub‐dendrogram is added to the dendrogram, the calculation is only based on the items that have already been in the dendrogram, while not on all the items in the matrix. This weakens the visualization power in some datasets if they only have intermediate levels of group‐wise differences. A pre‐splitting on heatmaps can greatly improve the distinguishability of group‐wise patterns. *ComplexHeatmap* provides various ways for splitting heatmap into “*slices*” into both rows and columns (Figure 2A,B): (1) Set a number of groups for *k*‐means clustering, where it also supports repeatedly running *k*‐means clustering to obtain a consensus *k*‐means clustering results to reduce the effect of randomness; (2) Set a categorical variable that contains predefined grouping information. The variable can be a vector or a data frame, then the heatmap is split by all combinations of levels in the categorical variable; (3) If the hierarchical clustering is already applied, the splitting can be specified as a single number so that cutree() is internally applied to cut the dendrogram. For the first two splitting methods, if clustering is turned on, hierarchical clustering is first performed within every heatmap slice, then a second clustering is applied over heatmap slices based on their means to show the slice‐level hierarchical relations.

As an example, the heatmap in Figure 2C includes genes that have significant differential expression in a glioblastoma cohort [25] with four subgroups (as the top annotation). The four subgroups are predicted by consensus clustering where the classification is very stable [26]. The stable classification is also supported by a *t*‐SNE analysis where the four subgroups are well separated (Supporting Information: Figure 1). However, if the hierarchical clustering is directly applied by pooling all samples, the four subgroups are not well separated as expected, whereas some samples in subgroups 3 (blue) and 4 (purple) are mixed. In Figure 2D, with the same matrix, columns are first split by the classification, then the hierarchical clustering is applied within each column slice separately. As a comparison, it indeed improves the visualization of group‐wise patterns. Also, in Figure 2D, rows are additionally split by *k*‐means clustering. Now it is very straightforward to observe the expression patterns of subgroup‐specific signature genes.

#### Render heatmap body as raster images

When we produce so‐called “high‐quality figures,” normally the figures are saved as vector graphics in the formats of, for example, pdf or svg. The vector graphics store the details of every single graphic element, thus if a heatmap made from a huge matrix is saved as vector graphics, the file size would be very big and the complete image would take a long time to be rendered by image viewers. Due to the limited size and resolution of the graphics device, neighboring grids from the heatmap are actually merged into single pixels for large heatmaps. Thus, it is not necessary to keep all the details of the huge heatmap and proper methods should be applied to efficiently reduce the original image.

Rasterization is a method to convert an image into a matrix of colors in red‐green‐blue (RGB) values. Let us assume a matrix for heatmap has *n*_*r*_ rows and *n*_*c*_ columns. When it is drawn on a certain graphics device, for example, an on‐screen device, the corresponding heatmap body uses *p*_*r*_ and *p*_*c*_ pixels for the rows and columns, respectively. When *n*_*r*_ > *p*_*r*_ and/or *n*_*c*_ > *p*_*c*_, multiple values in the matrix are mapped to single pixels where *n*_*r*_ and/or *n*_*c*_ can be reduced to be equal to *p*_*r*_ and/or *p*_*c*_. *ComplexHeatmap* provides three methods to reduce the graphics in the heatmap body by rasterization: (1) First the heatmap body is written as a temporary png image with *p*_*r*_ × *p*_*c*_ resolution, then the temporary image is read as a *raster* object and filled back to the heatmap body. In this way, the image reduction is performed on the png device. (2) The original matrix is first reduced to the size of *p*_*r*_ × *p*_*c*_ so that one single value in the reduced matrix can correspond to a distinct pixel. The reduction on the matrix can be applied with a specific method, such as taking the mean or a random value from the submatrices. (3) A temporary image with resolution *n*_*r*_ × *n*_*c*_ is first generated, then the *magick* package is used to reduce the image to the size *p*_*r*_ × *p*_*c*_, finally, the reduced image is read as a *raster* object and filled into the heatmap body. The *magick* package provides a large number of methods for resizing the image and they are all supported in *ComplexHeatmap*. In “Section 2.8 Heatmap as raster image” of the *ComplexHeatmap* book, there are detailed visual comparisons of different image reduction methods.

#### Customize heatmap

By default, heatmap bodies are composed of a two‐dimensional organization of colored grids or cells. *ComplexHeatmap* allows users to customize heatmap bodies by adding new layers of graphics. Arguments cell_fun and layer_fun in Heatmap() can be used to add self‐defined graphics to heatmap cells when heatmap is drawing (Figure 2A). The two arguments are basically the same except layer_fun is a vectorized version of cell_fun, which makes the drawing faster if the heatmap body is large. More generally, decorate_*() family functions, for example, decorate_annotation(), add graphics to any heatmap component after the heatmap has been drawn. Every heatmap component has its own plotting region (or viewport) and they are still recorded after the heatmap is drawn. decorate_*() can go back to a specific viewport and add self‐defined graphics there afterward.

As will be introduced in later sections, the 3D heatmap, the oncoPrint, and the UpSet plot are internally implemented with layer_fun; the density heatmap and enriched heatmap are partially enhanced by implementing decorate_heatmap_body().

#### Flexible controls of colors and legends

In a heatmap, colors are the major aesthetic elements mapping to data. *ComplexHeatmap* allows exact mapping between colors and values in the matrix by a color mapping function by specifying breaks and corresponding colors, then remaining colors are linearly interpolated in the corresponding intervals in a specific color space. For example, users can define a color mapping function that is symmetric to zero, which is useful for identifying the expression of up‐regulated and down‐regulated genes, or users can define the same color mapping functions for different heatmaps to make colors comparable between them. It also allows flexible configurations on heatmap legends, such as multi‐color scheme legends and legends with self‐defined graphics. We kindly refer readers to “Chapter 5. Legends” in the *ComplexHeatmap* book for more demonstrations.

### Heatmap annotations

**Figure 3:**
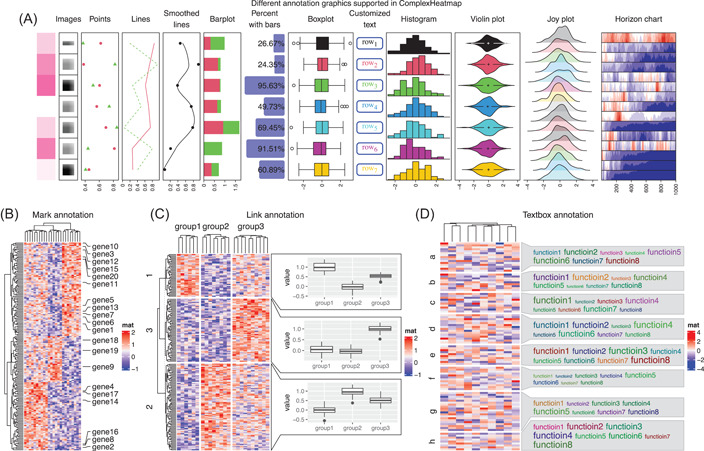
Demonstration of various heatmap annotation graphics. (A) Different annotation graphics are supported in *ComplexHeatmap*. (B) The mark annotation. (C) The link annotation. (D) The textbox annotation. Data in all four figures are randomly generated.

Heatmap annotations are important components of a heatmap. It not only shows additional information associated with heatmap rows and columns, but also allows visualizing with more types of graphics. *ComplexHeatmap* provides flexible support for built‐in annotations as well as new self‐defined annotation graphics. In Figure 3A, we demonstrate part of annotation graphics that are by default supported in *ComplexHeatmap* (from left to right):

1. Heatmap‐like annotation. It is called “simple annotation” in *ComplexHeatmap*. It visualizes a vector or a matrix, either in numeric or character.
2. Image annotation. It supports images in various formats, for example, png, svg, pdf, or jpg.
3. Points annotation. It supports a single numeric vector or a numeric matrix.
4. Lines annotation. It supports a single numeric vector or a numeric matrix.
5. Smoothed lines annotation. The smoothing is applied by the *loess* method.
6. Bar plot annotation. It supports stacked bar plots.
7. Percent annotation. It contains both text and bars.
8. Boxplot annotation.
9. Text annotation. It supports constructing customized text with the *gridtext* package.
10. Histogram annotation.
11. Violin annotation. It visualizes a list of distributions. Alternatively, the distributions can be visualized by normal density plots or heatmaps.
12. Joy plot annotation. The peaks can be extended into neighbors' plotting regions.
13. Horizon chart annotation. A horizon chart is a visualization method that vertically splits an area chart with uniform size, then the bands are layered on top of each other [27].

All built‐in annotation graphics are implemented by annotation functions named with anno_ prefix, for example, anno_points() for points annotation. Besides the above‐listed annotations, *ComplexHeatmap* supports more complex ones. For example, there is a “mark annotation” by anno_mark(), which draws labels for a subset of rows or columns where the labels are shifted from their original positions to get rid of overlapping, and lines are drawn to connect labels to their original rows or columns (Figure 3B). There is also a “link annotation” by anno_link(), which links plotting regions to subsets of a heatmap. The link annotation provides a general solution to associate more self‐defined graphics to correspond to heatmap rows or columns. In Figure 3C, three *ggplot2* plots are created that visualize distributions of values in the three column groups but only in the selected subsets of rows. In Figure 3D, a list of words is associated with each row group where the font sizes correspond to the importance of the words. This has been specifically implemented with the function anno_textbox(), and it has been used to summarize the biological functions of genes in the *simplifyEnrichment* package [28].

The constructor function HeatmapAnnotation() accepts multiple annotations specified as name‐value pairs. Simple annotations are specified as vectors, matrices, or a data frame. Other annotations should be specified via functions anno_*(). An example with four annotations is demonstrated as follows.

**Table:** 

ha = HeatmapAnnotation(
foo = runif(10),
bar = sample(letters[1:4], 10, replace = TRUE),
pt = anno_points(runif(10)),
txt = anno_text(month.name[1:10]))

Row annotations should be set with one additional argument which = "row" or with the helper function rowAnnotation(). *ComplexHeatmap* already provides a large number of annotation graphics, nevertheless, *ComplexHeatmap* provides an interface for creating self‐defined annotation graphics. We kindly refer readers to “Section 3.20 Implement new annotation functions” in the *ComplexHeatmap* book for more details.

### A list of heatmaps

The promising feature of *ComplexHeatmap* is that it supports concatenating multiple heatmaps and annotations so that it is possible to visualize associations between various sources of information. *ComplexHeatmap* provides a simple syntax for concatenating heatmaps with the operator +. The expression returns a *HeatmapList* object and directly printing the *HeatmapList* object draws the heatmap. An example usage is as follows:

**Table:** 

Heatmap(…)+
Heatmap(…)+
rowAnnotation(…)

We previously introduced annotations as components of a single heatmap. Here row annotations can also be independently concatenated to the heatmap list, as demonstrated in the code above. Alternatively, which is less used, the heatmap lists can be vertically concatenated with the operator %v%.

**Table:** 

Heatmap(…)%v%
Heatmap(…)%v%
HeatmapAnnotation(…)

The number of heatmaps and annotations to be concatenated can be arbitrary. The ordering and splitting of all heatmaps are adjusted by the main heatmap, which is by default the first numeric heatmap, or the other heatmap in the list that is specified by the user.

#### Visualize associations between DNA methylation and gene expression

**Figure 4:**
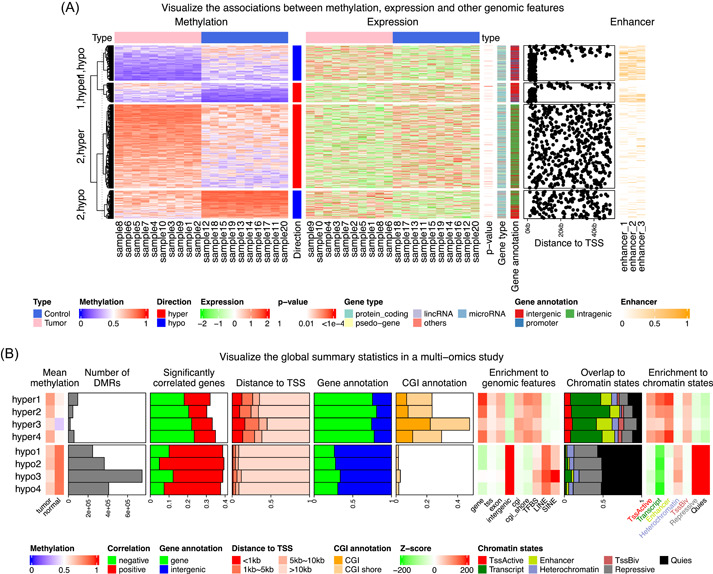
Demonstration of complex heatmap list visualization. (A) Visualization of the association between DNA methylation, gene expression, and related genomic features. For simplicity of the figure, it only includes DMRs showing negative correlations to associated genes. (B) Comprehensive visualization of the global summary statistics of an epigenomics study. DMRs, differentially methylated regions.

Figure 4A demonstrates a complex heatmap visualization on a dataset randomly generated but based on patterns found in unpublished work. It visualizes associations between DNA methylation, gene expression, enhancers, and gene‐related information. In heatmaps, each row corresponds to a differentially methylated region (DMR, which is a genomic region showing significantly different methylation between tumor and control samples) or other attributes associated with the corresponding DMR. In Figure 4A, there are the following heatmaps and annotations from left to right:

1. A heatmap of methylation in DMRs.
2. A one‐column heatmap showing the direction of differential methylation. “Hyper” means the methylation is higher in tumor samples and “hypo” means the methylation is lower in tumor samples.
3. A heatmap of gene expression. They are the nearest genes to the DMRs.
4. A one‐column heatmap of *p*‐values from the Pearson correlation test on methylation in DMRs and expression of associated genes.
5. A one‐column heatmap of the type of genes, for example, protein‐coding genes or lincRNAs?
6. A one‐column heatmap of the locations of DMRs, for example, in promoters or in intergenic regions?
7. A points annotation of the genomic distances between DMRs to transcription start sites (TSSs) of the associated genes.
8. A heatmap of the overlap between enhancers and DMRs. The value measures the fraction of a DMR covered by enhancers.

In Figure 4A, the heatmap list is split by the combination of directions of differential methylation and a two‐group *k*‐means clustering. The grouping by *k*‐means is to distinguish the high‐methylation and low‐methylation groups on rows. The complex heatmaps reveal that highly methylated DMRs are enriched in intergenic and intragenic regions, and they rarely overlap with enhancers (row groups “2,hypo” and “2,hyper”), while in contrast, lowly methylated DMRs are enriched in promoters and enhancers (row groups “1,hypo” and “1,hyper”). This might imply that enhancers associated with low methylation and methylation changes in enhancers might affect their transcriptional activities on related genes.

#### Visualize global summary statistics in multi‐omics studies

Multi‐omics studies integrate data from genomics, transcriptomics, or epigenomics to look for novel associations in biological systems from different levels. Thus, it is important to properly and effectively visualize the potential connections between these different data types. Figure 4B demonstrates one such typical landscape summary visualization where a list of different statistics based on single data types or combinations of multiple data types are aggregated by a list of heatmaps and annotation graphics. Figure 4B is based on a glioblastoma cohort study [29], which studies the epigenomic difference between four subtypes (indexed as 1 to 4 in Figure 4B) with DNA methylation, gene expression, and histone modification data. The study generates four sets of DMRs where each one compares methylation in one subtype against normal samples. Figure 4B visualizes various genomic attributes of DMRs with a list of heatmaps and annotations, additionally split by the direction of methylation change. From left to right in Figure 4B there are:

1. A heatmap of the mean methylation in DMRs in tumor samples and in normal samples.
2. A bar plot annotation of the number of DMRs in each category.
3. A stacked bar plot annotation of the fractions of DMRs that show significant correlations to the expression of the nearest genes.
4. A stacked bar plot annotation of the distances of DMRs to the TSSs of the nearest genes.
5. A stacked bar plot annotation of the fractions of DMRs that overlap to genes or intergenic regions.
6. A stacked bar plot annotation of the fractions of DMRs that overlap to CpG islands (CGIs) or CGI shores.
7. A heatmap of the enrichment of DMRs to a list of genomic features. A positive value means over‐representation. The enrichment test is applied by randomly shuffling DMRs in the genome by taking the Jaccard coefficient as the statistic. *z*‐scores (observed–expected)/(standard deviation) is used for the heatmap.
8. A stacked bar plot of the fractions of DMRs that overlap to chromatin states.
9. A heatmap of the enrichment of DMRs to the chromatin states. Similarly, *z*‐scores are used for the heatmap.

In the heatmap list in Figure 4B, it is straightforward to observe the different features of subgroup‐specific DMRs. For example, hyper‐DMRs have more negative correlations between methylation and gene expression, and hypo‐DMRs are located more in the intergenic regions and inactive chromatin states. To conclude, such visualization provides a powerful bird's‐eye view of the global attributes in a complex study.

### High‐level plots

The flexibility of *ComplexHeatmap* allows users to implement new high‐level graphics on data with matrix‐like structures. *ComplexHeatmap* has already implemented several high‐level graphics functions and they are introduced in the following subsections. Note all these functions are basically specific customizations on heatmaps. They are essentially in the *Heatmap* class and they can be concatenated to general heatmaps and annotations to form complex visualizations.

#### Density heatmap

**Figure 5:**
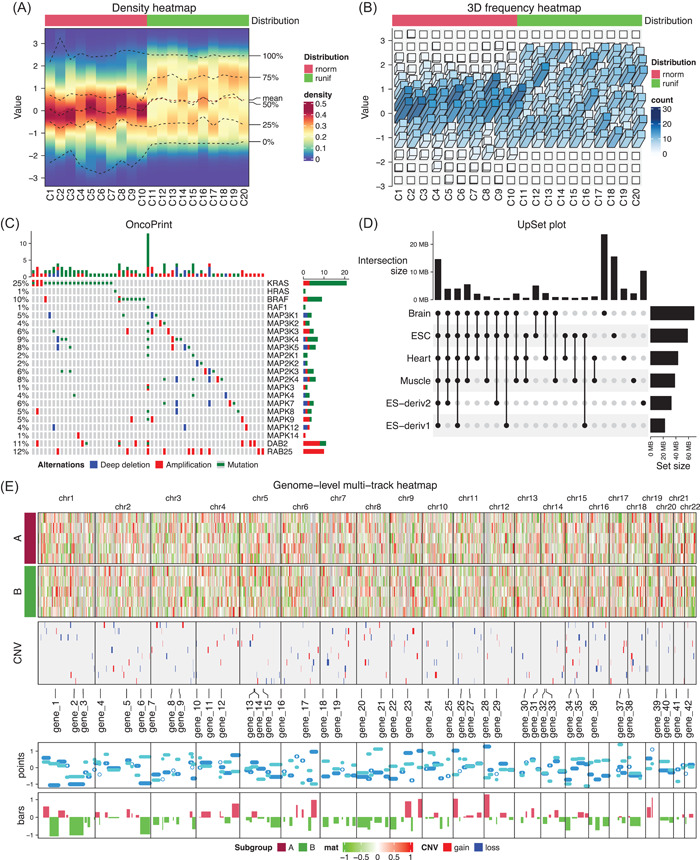
Demonstration of high‐level plots implemented in *ComplexHeatmap*. (A) The density heatmap. Values in the first 10 columns are generated from the normal distribution and values in the second 10 columns are generated from the uniform distribution. (B) 3D frequency heatmap. The input matrix is the same as in Figure (A). (C) The oncoPrint. The lung adenocarcinoma carcinoma dataset from cBioPortal is used. Only a subset of genes and patients are used due to the limited figure size. (D) The UpSet plot. The H3K4me3 ChIP‐seq peaks from six human tissues are from the Roadmap project. (E) Genome‐level multiple‐track plot. The data are randomly generated.

To visualize data distributions in a matrix or a list, normally boxplots or violin plots are used. However, when the number of distributions becomes large, boxes or violins would not be efficient visualization methods. The function densityHeatmap() uses colors to map the density values and it is able to visualize a large number of distributions (Figure 5A). In densityHeatmap(), the similarity between distributions can be measured with the Kolmogorov–Smirnov distance.

#### Three‐dimensional heatmap

Three‐dimensional (3D) visualization is in general not recommended for representing data [30], but it might be helpful for specific scenarios. *ComplexHeatmap* supports converting a normal heatmap to a 3D heatmap by converting heatmap grids to 3D bars, which are drawn as projections onto the two‐dimensional plate. 3D heatmaps are drawn with the function Heatmap3D() and it accepts the same set of arguments as Heatmap(). The density distributions in Figure 5A can also be visualized as a list of bar plots where bars are represented in 3D (Figure 5B). It is recommended to map both colors and bar heights to data in 3D heatmap visualization.

#### oncoPrint

The function oncoPrint() visualizes multiple genomic alteration events, for example, single‐base mutations (SNVs), fragment insertion or deletions (Indels), or copy number variations (CNVs), in a list of genes and in multiple patients. oncoPrint() provides a general solution where graphics for specific genome alterations can be self‐defined (Figure 5C). By default, genes are ordered by their total numbers of alterations, and patients are reordered to show mutual exclusivity in the cohort. As oncoPrint() returns a *Heatmap* object, it can be concatenated to heatmaps of other genomic datasets, for example, gene expression, to show more complicated genomic associations.

#### UpSet plot

The UpSet plot [31] provides a more efficient way to visualize intersections in a large number of sets compared to the traditional approach, that is, the Venn Diagram. The function UpSet() in *ComplexHeatmap* provides an enhanced implementation of the original tool [32]. Additionally, UpSet() is capable of working on intersections of genomic regions from multiple lists, which helps to reveal, for example, tissue‐specific chromatin modifications (Figure 5D).

#### Genome‐level plots

Genome‐level heatmaps are frequently used in genomics studies, for example, for visualizing the global copy number variation profiles [33]. The key to making genome‐level heatmaps is to bin the genome and to normalize various genomic signals to the genome bins to form matrices, then normal heatmap visualization can be applied to them. In Figure 5E, we demonstrated a genome‐level visualization with two heatmaps and multiple additional tracks, which are created as heatmap annotations.

### Integrated into other packages

#### EnrichedHeatmap

An enriched heatmap specifically visualizes the enrichment of a certain type of genomic signal on a list of genomic features of interest [34]. For example, how chromatin modifications are enriched around gene TSSs or how DNA is lowly methylated around CGIs. The *EnrichedHeatmap* package [6] is built upon *ComplexHeatmap* and it provides a general solution for such spatial relations of two types of genomic features. It also implements a special annotation function anno_enriched() that summarizes the average enrichment over all genomic features. Being unique to other similar tools, it is able to normalize categorical genomic signals such as chromatin segmentations. More importantly, the enriched heatmap is also a *Heatmap* object, thus it inherits all the features from the *Heatmap* class, such as heatmap splitting and concatenating to more heatmaps.

**Figure 6:**
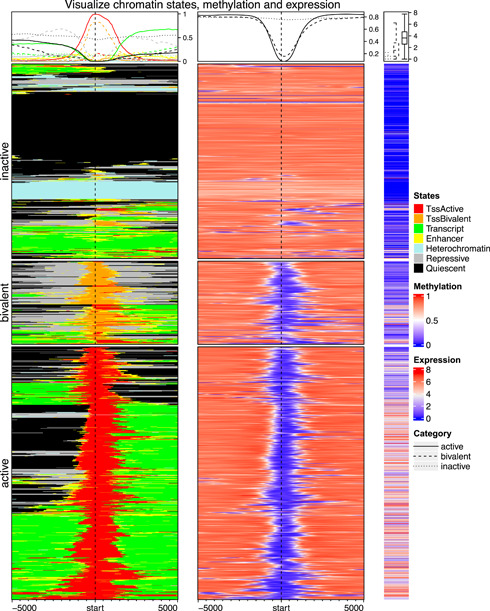
A list of enriched heatmaps and normal heatmaps. From the left to right are the heatmaps of chromatin states, DNA methylation, and gene expression. Data are from the Roadmap project.

Figure 6 demonstrates a complex visualization of the distribution of chromatin states and DNA methylation around gene TSSs, as well as the expression of associated genes. The data are from the Roadmap project [35]. Heatmaps are split into three groups where TSSs are in active states, bivalent states, and inactive states. Through the heatmaps, it can be easily observed that active TSSs are associated with low methylation and the corresponding genes are highly expressed. Bivalent TSSs, although are also lowly methylated, have a lower expression for the genes. In comparison, inactive TSSs are almost fully methylated and the expression of corresponding genes is normally silenced.

#### InteractiveComplexHeatmap

*ComplexHeatmap* only generates static plots. The companion package *InteractiveComplexHeatmap* [36] can seamlessly convert a static heatmap to an interactive Shiny application where users can interact with the heatmap, for example, by clicking and brushing over heatmaps. This is useful when specific patterns are observed on heatmaps, and detailed information about them can be easily extracted by directly interacting with the heatmaps. The conversion from static heatmaps to interactive ones can be easily done with the function htShiny(), which is called with no argument after the static heatmap is generated. This functionality works for all kinds of heatmaps, as long as they are generated by *ComplexHeatmap*. Moreover, *InteractiveComplexHeatmap* provides flexible solutions for customizing the user interface of the interactive heatmap applications as well as for defining the responses to users' actions on heatmaps.

## CONCLUSION

Complex heatmap visualization is a powerful way to associate multiple sources of information. In this article, we systematically demonstrated the rich functionalities of the *ComplexHeatmap* package. We believe *ComplexHeatmap* will continually be a useful tool for bioinformatics and the general data science field for revealing hidden structures in the data.

## AUTHOR CONTRIBUTIONS

**Zuguang Gu:** Conceptualization, Software, Visualization, Formal analysis, Validation, Investigation, Writing—Original Draft, Review & Editing. All authors conceived the project, and have read and approved the final manuscript.

## CONFLICT OF INTEREST

The author declares no conflict of interest.

## Supporting information

Supplementary information.

## Data Availability

The stable version of *ComplexHeatmap* is available at https://bioconductor.org/packages/ComplexHeatmap/. The development version of *ComplexHeatmap* is available at https://github.com/jokergoo/ComplexHeatmap. The documentation of *ComplexHeatmap* is available at https://jokergoo.github.io/ComplexHeatmap‐reference/book/. The source code for producing figures in this article is available at https://github.com/jokergoo/ComplexHeatmap_v2_paper_code. Supplementary materials (figures, tables, scripts, graphical abstract, slides, videos, Chinese translated version, and update materials) may be found in the online DOI or iMeta Science http://www.imeta.science/.

## References

[1] Heat map. Wikipedia. https://en.wikipedia.org/wiki/Heat_map.

[2] Wilkinson, Leland , and Michael Friendly . 2009. “The History of the Cluster Heat Map.” The American Statistician 63: 179–84. 10.1198/tas.2009.0033.

[3] Eisen, Michael B. , Paul T. Spellman , Patrick O. Brown , and David Botstein . 1998. “Cluster Analysis and Display of Genome‐Wide Expression Patterns.” Proceedings of the National Academy of Sciences of the United States of America 95: 14863–69. 10.1073/pnas.95.25.14863. 9843981 PMC24541

[4] Wolff, Joachim , Rabbani Leily , Ralf Gilsbach , Gautier Richard , Thomas Manke , Rolf Backofen , and Björn A Grüning . 2020. “Galaxy HiCExplorer 3: A Web Server for Reproducible Hi‐C, Capture Hi‐C and Single‐Cell Hi‐C Data Analysis, Quality Control and Visualization.” Nucleic Acids Research 48: W177–84. 10.1093/nar/gkaa220 32301980 PMC7319437

[5] Hahne, Florian , and Robert Ivanek . 2016. “Visualizing Genomic Data Using Gviz and Bioconductor.” In Statistical Genomics, edited by Ewy Mathé and Sean Davis, 335–51. 10.1007/978-1-4939-3578-9_16. 27008022

[6] Gu, Zuguang , Roland Eils , Matthias Schlesner , and Naveed Ishaque . 2018. “EnrichedHeatmap: An R/Bioconductor Package for Comprehensive Visualization of Genomic Signal Associations.” BMC Genomics 19: 234. 10.1186/s12864-018-4625-x. 29618320 PMC5885322

[7] Gu, Zuguang , Lei Gu , Roland Eils , Matthias Schlesner , and Benedikt Brors . 2014. “Circlize Implements and Enhances Circular Visualization in R.” Bioinformatics 30: 2811–12. 10.1093/bioinformatics/btu393. 24930139

[8] Gu, Zuguang , and Daniel Hübschmann . 2021. “Spiralize: An R Package for Visualizing Data on Spirals.” Bioinformatics 38: 1434–36. 10.1093/bioinformatics/btu393. PMC8826351 34849585

[9] Gu, Zuguang , Roland Eils , and Matthias Schlesner . 2016. “HilbertCurve: An R/Bioconductor Package for High‐Resolution Visualization of Genomic Data.” Bioinformatics 32: 2372–74. 10.1093/bioinformatics/btw161. 27153599

[10] Wickham, Hadley . 2009. ggplot2: Elegant Graphics for Data Analysis. New York: Springer New York. 10.1007/978-0-387-98141-3.

[11] Gaujoux, Renaud , and Cathal Seoighe . 2010. “A Flexible R Package for Nonnegative Matrix Factorization.” BMC Bioinformatics 11: 367. 10.1186/1471-2105-11-367. 20598126 PMC2912887

[12] Barter, Rebecca L. , and Yu Bin . 2018. “Superheat: An R Package for Creating Beautiful and Extendable Heatmaps for Visualizing Complex Data.” Journal of Computational and Graphical Statistics 27: 910–22. 10.1080/10618600.2018.1473780. 30911216 PMC6430237

[13] Zhao, Shilin , Yan Guo , Quanhu Sheng , and Yu Shyr . 2014. “Heatmap3: An Improved Heatmap Package with More Powerful and Convenient Features.” BMC Bioinformatics 15: P16. 10.1186/1471-2105-15-S10-P16.

[14] Gu, Zuguang , Roland Eils , and Matthias Schlesner . 2016. “Complex Heatmaps Reveal Patterns and Correlations in Multidimensional Genomic Data.” Bioinformatics 32: 2847–49. 10.1093/bioinformatics/btw313. 27207943

[15] Wang, Liang‐Bo , Alla Karpova , Marina A. Gritsenko , Jennifer E. Kyle , Song Cao , Yize Li , Dmitry Rykunov , et al. 2021. “Proteogenomic and Metabolomic Characterization of Human Glioblastoma.” Cancer Cell 39: 509–28.e20. 10.1016/j.ccell.2021.01.006. 33577785 PMC8044053

[16] Lucas, Carolina , Patrick Wong , Jon Klein , Tiago B. R. Castro , Julio Silva , Maria Sundaram , Mallory K. Ellingson , et al. 2020. “Longitudinal Analyses Reveal Immunological Misfiring in Severe COVID‐19.” Nature 584: 463–69. 10.1038/s41586-020-2588-y. 32717743 PMC7477538

[17] Masuda, Takahiro, Roman Sankowski, Ori Staszewski, Chotima Böttcher, Lukas Amann, Sagar, Christian Scheiwe, et al. 2019. “Spatial and Temporal Heterogeneity of Mouse and Human Microglia at Single‐Cell Resolution.” *Nature* 566: 388–92. 10.1038/s41586-019-0924-x. 30760929

[18] Dufva, Olli , Petri Pölönen , Oscar Brück , Mikko A. I. Keränen , Jay Klievink , Juha Mehtonen , Jani Huuhtanen , et al. 2020. “Immunogenomic Landscape of Hematological Malignancies.” Cancer Cell 38: 380–99.e13. 10.1016/j.ccell.2020.06.002. 32649887

[19] Tragin, Margot , and Daniel Vaulot . 2018. “Green Microalgae In Marine Coastal Waters: The Ocean Sampling Day (OSD) Dataset.” Scientific Reports 8: 14020. 10.1038/s41598-018-32338-w. 30232358 PMC6145878

[20] Hu, Anyi , Shuang Li , Lanping Zhang , Hongjie Wang , Jun Yang , Zhuanxi Luo , Azhar Rashid , et al. 2018. “Prokaryotic Footprints in Urban Water Ecosystems: A Case Study of Urban Landscape Ponds in a Coastal City, China.” Environmental Pollution 242: 1729–39. 10.1016/j.envpol.2018.07.097. 30064876

[21] Galili, Tal . 2015. “dendextend: An R Package for Visualizing, Adjusting and Comparing Trees of Hierarchical Clustering.” Bioinformatics 31: 3718–20. 10.1093/bioinformatics/btv428. 26209431 PMC4817050

[22] Sakai, Ryo , Raf Winand , Toni Verbeiren , Andrew Vande Moere , and Jan Aerts . 2014. “Dendsort: Modular Leaf Ordering Methods for Dendrogram Representations in R.” F1000Research 3: 177. 10.12688/f1000research.4784.1. 25232468 PMC4162509

[23] Hahsler, Michael , Kurt Hornik , and Christian Buchta . 2008. “Getting Things in Order: An Introduction to the R Package Seriation.” Journal of Statistical Software 25: 1– 34. 10.18637/jss.v025.i03

[24] Kaiser, Sebastian , and Friedrich Leisch . 2008. “A Toolbox for Bicluster Analysis in R”. Department of Statistics: Technical Reports, No.28. 10.5282/ubm/epub.3293.

[25] Verhaak, Roel G. W. , Katherine A. Hoadley , Elizabeth Purdom , Victoria Wang , Yuan Qi , Matthew D. Wilkerson , C. Ryan Miller , et al. 2010. “Integrated Genomic Analysis Identifies Clinically Relevant Subtypes of Glioblastoma Characterized by Abnormalities in PDGFRA, IDH1, EGFR, and NF1.” Cancer Cell 17: 98–110. 10.1016/j.ccr.2009.12.020. 20129251 PMC2818769

[26] Gu, Zuguang , Matthias Schlesner , and Daniel Hübschmann . 2021. “cola: An R/Bioconductor Package for Consensus Partitioning Through a General Framework.” Nucleic Acids Research 49: e15. 10.1093/nar/gkaa1146. 33275159 PMC7897501

[27] Heer, Jeffrey , Nicholas Kong , and Maneesh Agrawala . 2009. “Sizing the Horizon: The Effects of Chart Size and Layering on the Graphical Perception of Time Series Visualizations.” In *CHI '09: Proceedings of the SIGCHI Conference on Human Factors in Computing Systems*, 1303–12. 10.1145/1518701.1518897.

[28] Gu, Zuguang , and Daniel Hübschmann . 2022. “Simplify Enrichment: A Bioconductor Package for Clustering and Visualizing Functional Enrichment Results.” *Genomics Proteomics Bioinformatics*. 10.1016/j.gpb.2022.04.008. PMC10373083 35680096

[29] Wu, Yonghe , Michael Fletcher , Zuguang Gu , Qi Wang , Barbara Costa , Anna Bertoni , Ka‐Hou Man , et al. 2020. “Glioblastoma Epigenome Profiling Identifies SOX10 as a Master Regulator of Molecular Tumour Subtype.” Nature Communications 11: 6434. 10.1038/s41467-020-20225-w. PMC7749178 33339831

[30] Wilke, Claus O. 2019. Fundamentals of Data Visualization. Sebastopol: O'Reilly Media.

[31] Lex, Alexander , Nils Gehlenborg , Hendrik Strobelt , and Hanspeter Pfister Romain Vuillemot . 2014. “UpSet: Visualization of Intersecting Sets.” IEEE Transactions on Visualization and Computer Graphics 20: 1983–92. 10.1109/TVCG.2014.2346248. 26356912 PMC4720993

[32] Conway, Jake R. , Alexander Lex , and Nils Gehlenborg . 2017. “UpSetR: An R Package for the Visualization of Intersecting Sets and Their Properties.” Bioinformatics 33: 2938–40. 10.1093/bioinformatics/btx364. 28645171 PMC5870712

[33] Wang, Rujin , Dan‐Yu Lin , and Yuchao Jiang . 2020. “SCOPE: A Normalization and Copy‐Number Estimation Method for Single‐Cell DNA Sequencing.” Cell Systems 10: 445–52.e6. 10.1016/j.cels.2020.03.005. 32437686 PMC7250054

[34] Ramírez, Fidel , Friederike Dündar , Sarah Diehl , Björn A. Grüning , and Thomas Manke . 2014. “deepTools: A Flexible Platform for Exploring Deep‐Sequencing Data.” Nucleic Acids Research 42: W187–91. 10.1093/nar/gku365. 24799436 PMC4086134

[35] Roadmap Epigenomics Consortium , Kundaje, Anshul , Wouter Meuleman , Jason Ernst , Misha Bilenky , Angela Yen , Alireza Heravi‐Moussavi , et al. 2015. “Integrative Analysis of 111 Reference Human Epigenomes.” Nature 518: 317–30. 10.1038/nature14248. 25693563 PMC4530010

[36] Gu, Zuguang , and Daniel Hübschmann . 2021. “Make Interactive Complex Heatmaps in R.” Bioinformatics 38: 1460–62. 10.1093/bioinformatics/btab806. PMC8826183 34864868

