# Supplementary material for: Complex heatmap visualization
Source: Imeta. 2022 Aug 1;1(3):e43. doi: 10.1002/imt2.43 (PMC10989952; doi:10.1002/imt2.43)
Supplement: Supplementary file 1 — Supplementary information. [file IMT2-1-e43-s001.docx]

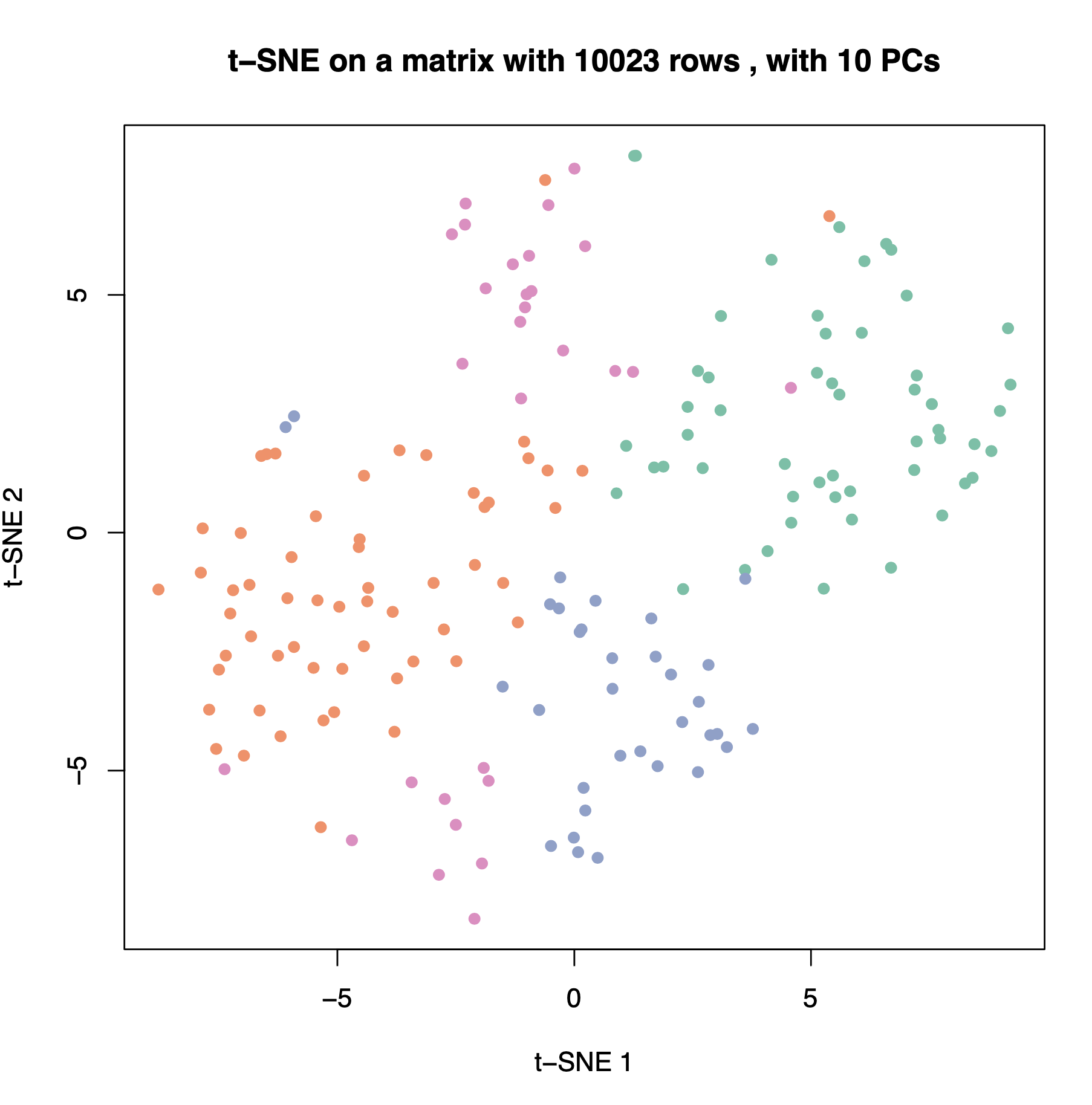


**Supplementary Figure 1.** The *t*-SNE visualization on the matrix used in Figure 2C-D. Colors correspond to the classifications from the consensus clustering.
